# Characterization of Silica Exposure during Manufacturing of Artificial Stone Countertops

**DOI:** 10.3390/ijerph17124489

**Published:** 2020-06-22

**Authors:** Mariella Carrieri, Carly Guzzardo, Daniel Farcas, Lorenzo G. Cena

**Affiliations:** 1 Department of Cardiologic, Thoracic Vascular Sciences and Public Health, University of Padua, 35128 Padua, Italy; mariella.carrieri@unipd.it; 2 Department of Health, West Chester University of Pennsylvania, West Chester, PA 19383, USA; CG803928@wcupa.edu; 3 Department of Occupational and Environmental Health Sciences, West Virginia University, Morgantown, WV 26505, USA; dfarcas@mix.wvu.edu; 4 Windjammer Environmental LLC, National Harbor, Washington, DC 20745, USA

**Keywords:** silica, crystalline, countertops, benchtops, manufacturing, grinding, respirable

## Abstract

Artificial stone is increasing in popularity in construction applications, including commercial and residential countertops. Eco-friendliness, durability, and resistance to staining, make artificial stone attractive to consumers. Health concerns have arisen during manufacturing of artificial stone due to increased incidence of silicosis after relatively short exposure. Three artificial stone samples (A, B, and C) and one natural granite sample were subjected to cutting and grinding in a controlled environment. Gravimetric analysis, X-Ray diffraction, and scanning electron microscopy with energy dispersive spectroscopy were employed to determine crystalline silica concentrations and particle morphology of bulk and respirable particles. Silica content of bulk dust from artificial samples A and B was 91%, sample C was <10%, while granite was 31%. Silica percent in the respirable fraction for samples A and B was 53% and 54%, respectively, while sample C was <5% and granite was 8%. Number concentrations for samples A and B were mainly in the nano-fraction, indicating potential for translocation of silica particles to other organs outside of the lungs. Respirable dust concentrations inside the chamber were well above Occupational Safety and Health Administration standards for all materials, indicating that confined-space exposures require ventilation to lower risks of acute silicosis regardless of the nature of the stone.

## 1. Introduction

Silica, also known as silicon dioxide (SiO_2_), is a colorless, white chemical compound made of common elements which naturally occur in the earth’s crust. Silicon dioxide is the main component of more than 95% of rocks, hence many construction materials, such as concrete, sand, mortar, and stone, contain silicon dioxide in the form of crystalline silica; these materials are used in the fabrication of consumer products such as glass, ceramics, pottery, bricks, and artificial stone [1]. Silica is polymorphic, thus the same chemical formula of SiO_2_ can exist in more than one form of crystal structure. Silica in the form of quartz is the second most abundant mineral on the earth’s crust (the most abundant mineral is feldspar); the other main polymorphs of silica are tridymite and cristobalite [2].

Respirable crystalline silica (particles with diameters smaller than 10 μm) is created during mechanical manipulations (e.g., cutting, grinding, polishing, and crushing) of stone, rock, concrete, brick, and mortar [3]. Historically, workers have been exposed to silica in natural environments during mining operations, tunnel excavations and quarry work [1]. Workers who inhale respirable crystalline silica particles are at an increased risk of developing serious silica-related diseases, including silicosis, lung cancer, chronic obstructive pulmonary disease, and kidney disease [4]. In recent years, cases of silicosis have been reported among slate pencil workers [5], stonemasons [6,7,8,9], metal grinders [10], denim sandblasting [11,12,13], dental supplies manufacturing [14], and manufacturing of artificial stone countertops [15,16,17,18,19,20,21,22,23]. About 2.3 million workers in the U.S. are exposed to silica at work [1].

Chronic silicosis is a progressive and incurable occupational lung disease caused by the inhalation of respirable crystalline silica dust and can progress to respiratory failure and death [24]. Silicosis is marked by scarring and thickening of the lung tissue and no effective specific treatment is available for the disease; patients are provided supportive care, and some patients may be considered for lung transplant. Chronic silicosis can develop following decades of exposure to silica dust and can progress even after occupational exposure has ceased; the typical latency period of chronic silicosis is over 20 years [24]. High-intensity silica exposure has been associated with acute and accelerated silicosis. Acute silicosis may develop within a few weeks to less than 5 years of high-intensity exposure [3,25]. Accelerated silicosis develops within 10 years of moderate–high levels of exposure [24].

In China, between 1991 and 1995 over 500,000 cases of silicosis were reported [26]. In South Africa between 1975 and 2013, more than 10,000 deaths from silicosis were reported among miners [27]. A marked increase in silicosis cases has been reported in Australia, where 98 cases were detected in 2019 alone, with news outlets calling the phenomenon an “epidemic worse than asbestos” [28]. Following similar patterns, silicosis cases increased from 95 to 295 in Spain between 2003 and 2011 [15], and 82 new cases occurred in Israel from 1997 to 2015 [20]. In the U.S., the number of deaths from silicosis peaked in 1968 and started declining afterwards [29]. Recently, however, hazardous silica exposures have increased again. Between 2001 and 2010, a total of 1437 deaths had silicosis coded as an underlying or contributing cause of death [30]. Newly documented cases of silicosis have been reported during hydraulic fracturing (fracking) of gas and oil wells [31] and during fabrication and installation of artificial stone countertops [16,32].

Artificial stone countertops contain natural stone, quartz, resins, and Portland cement and a combination of these materials can contain more than 90% silica crystals [33]. In contrast, natural stone contains median values of 50% or less silica crystals [31]. Artificial stone has several desirable properties over natural stone: it is lighter and thinner than natural stone, it is non-porous and therefore has strong mechanical resistance [34]. Countertop manufacturers are marketing the new material as stain and scratch proof, acid and fire resistant, environmentally friendly, and non-toxic [35]. Aesthetically these artificial materials are almost indistinguishable from natural stone [36]. Because of its desirable properties, artificial stone has become a common choice in homes for kitchen or bathroom countertops as well as construction material in public buildings such as bars, restaurants, and store counters.

In spite of the use of wet-cutting methods and development of dust-suppression systems aimed at controlling dust exposure during manufacturing of artificial countertops, [37,38] new outbreaks of silicosis have been reported in the U.S. [39], Spain [15,40], Israel [16], Australia [22], and Italy [33] among workers making countertops from quartz artificial stone. In one study in Italy, seven silicosis cases were reported in a group of 29 workers, a prevalence of 25% [33]. The studies in Spain have reported even higher prevalence of 54% [15]. Moreover, silicosis has been reported in young subjects, with a median age of 29 years old and with relatively short seniority, often needing lung transplants [40].

Because of the widespread use of new artificial materials, operations, and tasks placing workers at risk for silicosis, efforts to limit workplace exposure to crystalline silica need to be maintained. The purpose of this research was to evaluate the chemical and physical properties of the dust generated during countertop fabrication by simulating a cutting and grinding process in a controlled environment. Controlled laboratory experiments exclude exposures from sources not directly associated with the materials in question and allow for accurate chemical analysis of the composition of the released particles. This study allows size segregation of the released particles and measurement of silica content in the respirable fraction associated with deeper particle penetration in the alveolar area of the lungs.

There are several processes and tasks that take place during countertop fabrication. The manufacturing tasks involving use of hand-held power tools include cutting, grinding, polishing, and laminating. Of these tasks, field studies have reported that cutting and grinding generate significantly higher crystalline silica exposures [38] and were therefore assessed in this study. 

## 2. Methods

### 2.1. Particle Generation

**Figure 1:**
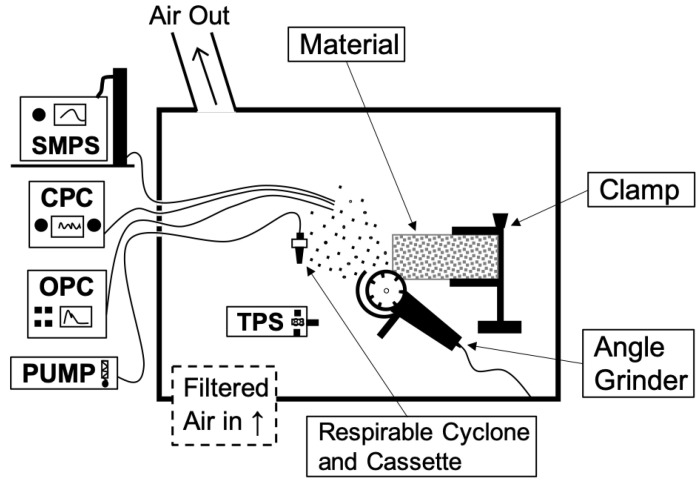
Experimental apparatus schematics. SMPS, scanning mobility particle spectrometer; CPC, condensation particle counter; OPC, optical particle counter; TPS, thermophoretic sampler; PUMP, air pump.

The experimental setup to simulate cutting and grinding is shown in Figure 1. The generation chamber measured 132 (width) by 163 (height) by 61 (depth) cm.

The chamber consisted of an adapted gloved cabinet with a clear window developed to accommodate and simulate various manufacturing processes. Inside the chamber an 11,000 rpm angle grinder (Model PC750AG Type 2; Porter Cable, Jackson, TN, USA) equipped with a 10-cm diamond segmented, stone-cutting blade (Model 50501-038; Norton Saint-Gobain, Worcester, MA, USA) was manually operated through gloves. The quartz countertop sample was held by a clamp and the grinder was used to perform 3.2 mm wide cuts along the edge of the sample.

Filtered, clean air was provided inside the chamber through high-efficiency particulate air filters and exhausted through a local exhaust ventilation system operated at a flowrate of 4060 L/min. 

### 2.2. Sample Materials

Three artificial stone countertop samples (materials A, B, and C) and one natural granite sample were examined in this study. The sample materials and dimensions are summarized in Table 1. The material specifications were provided by the manufacturers. Material A was composed of inorganic mineral fillers (85–95%), which included sand, cristobalite, silicon, glass, quartz, and ceramic particles that vary in proportions. This material also contained polymerized polyester resin (5–15%), while the remaining <5% was composed of additives and pigments. Material B contained quartz (>85%) and various resin and color pigments (<15%). Material C was produced by the sintering at high temperatures of various compacted materials, primarily aluminosilicates (clays, feldspars), silica (amorphous and crystalline), zircon, and <7% inorganic pigments. Following the sintering, the main crystalline mineral phases in the material are quartz and mullite and depending on the product, zircon, hematite, corundum, and anorthite/albite. No information was available on the composition of the natural granite sample.

### 2.3. Particle Sampling and Characterization

Direct-read instruments (DRIs) were positioned outside of the chamber and drew the particle-laden aerosol through sampling lines made of conductive silicon tubing (Figure 1). The inlets of the sampling lines were located at 20 cm from the samples and the length of the lines was 90 cm. A scanning mobility particle spectrometer (SMPS; Model 5.400; Grimm Technologies, Atlanta, GA, USA) at a flow rate of 0.6 L/min was used to measure particle number concentrations and mass concentrations that ranged from 20 nm to 350 nm. An optical particle counter (OPC; Model 11C; Grimm Technologies, Atlanta, GA, USA) at a flow rate of 1.2 L/min was used to measure particle number and mass concentrations ranging from 0.25 μm to 32 μm. A condensation particle counter (CPC; Model 3007; TSI, Inc., Shoreview, MN, USA) at a flow rate of 0.7 L/min was used to monitor total number concentration of particles from 0.01 μm to >1 μm to ensure proper operation of the chamber’s filtration system and determine when it was safe for the operator to open the chamber. 

Respirable samples were collected using a respirable cyclone (Model 225-01-02; SKC, Inc., Eighty Four, PA, USA) connected to a three-piece, 37 mm cassette with a 5 μm polyvinyl chloride (PVC) membrane filter (Model GLA-5000; Omega Specialty Instruments, Co., SKC, Kansas City, MO, USA) connected to a sampling pump (Model 220-5000TC; SKC Inc., Eighty Four, PA, USA) operating at 2.5 L/min. In addition, a thermophoretic sampler (TPS, Model TPS100; RJ Lee Group, Monroeville, PA, USA) collected particles directly on a transmission electron microscope (TEM) grid at a flow rate of 5 mL/min. The TPS was used to collect airborne particles directly on electron microscopy grids. A collection vessel made of aluminum foil was placed under the samples to collect particle debris from the grinding process. 

### 2.4. Sampling Protocol

Background measurements inside the closed sampling chamber were taken for 5 min with the DRIs when no grinding occurred. This allowed sufficient time for the instruments to provide at least two size-distribution scans. During background measurements, the angle grinder, TPS, and the respirable filter’s sampling pump were off. Following the background measurements, the angle grinder was turned on and the TPS and sampling pump were activated when the grinding started. Measurements were recorded with the DRIs for 7 min of continuous cutting and grinding. All experiments were performed in triplicate runs for all materials. New filter cassettes were used for each run. For each material, the TEM grid in the TPS was replaced after the first run (7 min total) while runs 2 and 3 were collected on the same substrate (14 minutes total). This was done to ensure sufficient time was provided for particle collection on the TPS substrate per material.

Between each change of material, the chamber was thoroughly washed and wiped to avoid cross-contamination. A new grinder with a new diamond blade was used for each material. Grinders, blades, and material samples were wiped with premoistened, deionized water wipes (Item 2903J75, Thomas Scientific, Swedesboro, NJ, USA) prior to use.

A field blank run was also performed where all protocols were repeated as during a normal sampling run with all instruments operating, including the angle grinder, with the exception that there was no contact between the grinder and the sample materials. This was done to determine the portion of particle generation from the grinder’s motor, which was subsequently subtracted from DRI’s concentration measurements.

### 2.5. Data Analysis

The DRIs’ data resulting from the 7 min sampling were averaged for the triplicate runs of each material. Background and field blank measurements were subtracted from the final measurements to account for background concentrations and particles generated by the grinder’s motor. Respirable mass (RM) concentrations were calculated from the OPC concentrations using the following equation:

(1) $$\mathrm{RM}=\sum_{i=1}^{15}\frac{\pi }{6}d_{OPC,\;i}^{3}\rho N_{OPC,i}S_{r}(d_{OPC,i})$$

where *d_OPC,i_* is the midpoint diameter of the OPC channel *i*, ρ is the particle density of the base material, *N_OPC,i_* is the number concentration designated by the OPC for a given size channel *i*, and *S_r_* is a function for the fraction of respirable mass [41].

Particle size distribution graphs were obtained from combined SMPS and OPC measurements. The OPC’s aerodynamic diameter was converted to the corresponding physical diameter using a shape factor of 1.35 for quartz [42]; subsequently, a linear interpolation method was used to avoid discontinuities of particle concentrations between the maximum and minimum cut-off diameter of SMPS and OPC, respectively. The midpoint diameter channels that ranged from 20 nm to 247.9 nm were used from the SMPS, while the midpoint diameter channels that ranged from 250 nm to 32,000 nm (32 µm) were used from the OPC.

The CPC was used for monitoring chamber leaks before and after grinding and to determine when it was safe to open the chamber to avoid operator’s exposure to silica. The CPC data was not further analyzed.

The number and respirable mass concentrations were averaged from the triplicate runs for each material and standard deviation of the three runs was obtained. One-way analysis of variance (ANOVA) tests were performed to assess differences between the three materials. Our hypothesis was that there would be no statistically significant differences between materials, and we anticipated that silica concentrations would be above those enforced by the Occupational Safety and Health Administration (OSHA) due to the enclosed nature of the experiments. All particle number and respirable mass concentrations were tested for normality before and after a log transformation using the Shapiro–Wilk test in the statistical analysis program R (version 3.6.1; The R Foundation for Statistical Computing, Vienna, Austria). Not all data passed the Shapiro–Wilk test for normality, therefore the values were also analyzed by the nonparametric Kruskal–Wallis test and Mood’s Median test.

Respirable filters were analyzed gravimetrically following the National Institute for Occupational Safety and Health (NIOSH) method 0600 and for crystalline silica by X-ray powder diffraction (XRD) following NIOSH method 7400 [43]. A portion of the bulk particle debris collected were mixed with calcium fluoride (CaF_2_) as an internal standard, ground further, and backloaded into a standard XRD holder. A separate portion of each sample was sieved to remove the portion of the sample larger than 45 µm. This was done to provide particle sizes appropriate to perform electron microscopy analysis on a polycarbonate filter since particles larger than 45 µm do not adhere well to the filters. The portion smaller than 45 µm was then mixed with the internal standard. The samples were scanned using standard run parameters on a diffractometer (X’Pert Pro, Panalytical Ltd, Malvern, UK) equipped with copper radiation. The weight percentage of silica was calculated through the use of the internal standard and calibration coefficients derived from standards NBS–1878a quartz, NBS–1879a cristobalite, and NIOSH/IITRI TY 27 tridymite mixed with CaF_2_. A portion of the unground material was examined by computer-controlled scanning electron microscopy (CCSEM) to determine particle sizing. The percentage respirable quartz was determined by multiplying the appropriate size fraction by the percentage quartz determined by XRD.

The TPS samples were manually examined in a high-resolution, field-emission scanning electron microscope with scanning transmission electron microscopy capabilities and equipped with X-ray diffraction (FESEM/STEM; S-5500; Hitachi High Technologies America, Schaumburg, IL, USA).

## 3. Results and Discussion

### 3.1. Direct-Read Instruments Analysis

**Figure 2:**
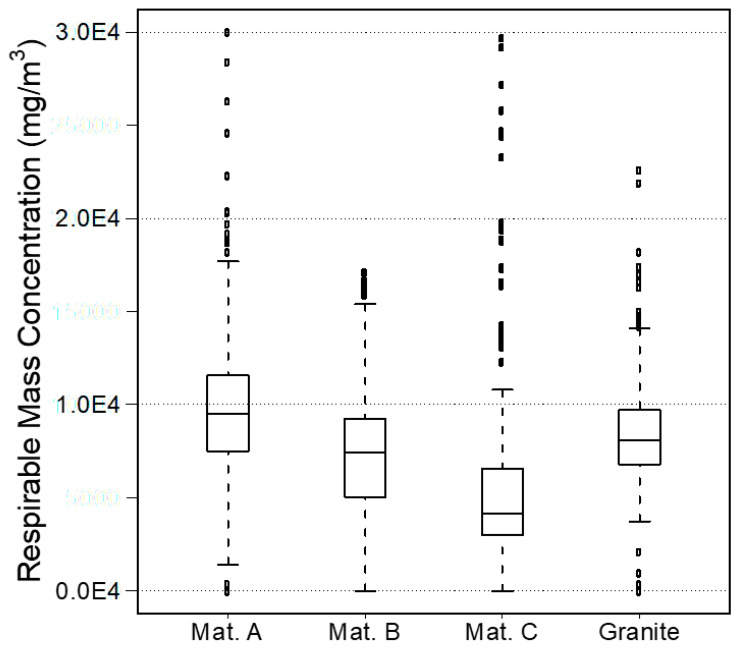
Boxplots of respirable mass concentrations by material.

**Figure 3:**
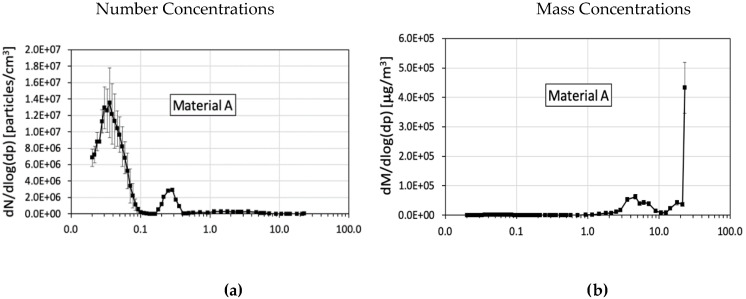

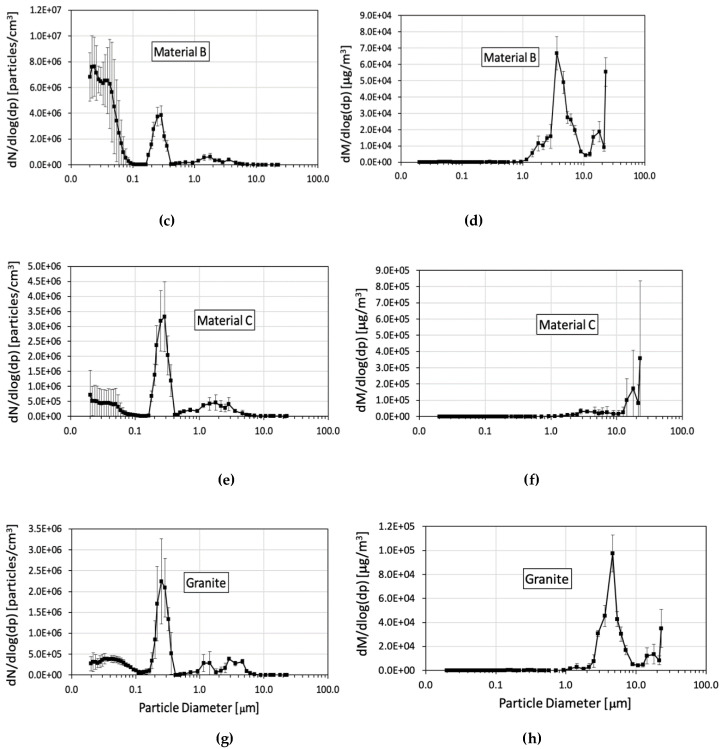
Average particle size distributions for all materials. Particle distributions by number are presented in the left column panels (**a,c,e,g**), Material A and Material B had a main peak in the nanometer range, between 0.01 and 0.1 μm, and a secondary peak between 0.15 and 0.4 μm (**a,c**). The main number concentration peak for Material C and for Granite was located between 0.15 and 0.4 μm, and the secondary peak was between 0.01 and 0.1 μm (**e,g**). Particle distributions by mass are presented in the right column panels (**b,d,f,h**) and were between 3 and 10 μm and above 20 μm for Material A (**b**). For Material B, the main peak was located between 1.0 and 10 μm (**d**). The main mass peak of Material C was above 10 μm (**f**) while for granite it was between 0.2 and 10 μm (**h**). Error bars represent standard deviation of three runs.

The results of the direct-read instruments for all materials tested are summarized in the boxplots in Figure 2 and particle size distributions in Figure 3. The upper and lower boundaries of the boxplots represent the 75th and 25th percentiles, the central line represents the median, the whiskers above and below represent the maximum and minimum values, while the individual dots represent outliers. Respirable mass concentrations were highest for material A (mean 9.78 × 10^3^ mg/m^3^, median 9.53 × 10^3^ mg/m^3^), followed by granite (mean 8.51 × 10^3^ mg/m^3^, median 8.07 × 10^3^ mg/m^3^), then material B (mean 7.34 × 10^3^ mg/m^3^, median 7.45 × 10^3^ mg/m^3^), and the lowest concentrations were recorded for material C (mean 6.22 × 10^3^ mg/m^3^, median 4.14 × 10^3^ mg/m^3^; Figure 2).

Due to the enclosed nature of the experiments, respirable mass concentrations were extremely high inside the chamber. The lower concentrations quantified while grinding material C may be due to the physical properties of this material. Material C was considerably harder to cut compared to the other three materials and required the operator to apply more force on the grinder to perform a cut. Noticeable sparks were generated when the blade of the grinder first came in contact with the sample, and the debris contained more of the large (a few mm in diameter) fragments than the other materials. While the lower respirable mass concentrations are a desirable outcome in terms of workers’ exposure, the material’s toughness may be undesirable in terms of difficulty to work with during manufacturing and installations. Material B was considerably thinner (0.6 cm) than the others (ranging between 1.15 and 2.7 cm), meaning that less of material B came in contact with the blade during each cut. Respirable mass concentrations measured by the instruments for material B, however, were comparable to those generated by the granite sample which was more than 4-times thicker (2.7 cm). No leaks were detected in the experimental chamber as per the CPC instrument. 

The results of the statistical analysis are reported in Table 2. Our hypothesis was that there would be no statistically significant differences between materials. All tests indicate that the null hypothesis should be rejected and that the means and medians are not all equal. 

The size distribution graphs in Figure 3 show the number and mass concentration values by particle size. All materials presented multimodal size distributions both in the number and mass concentrations. In general, material A produced higher particle number and mass concentrations than the other materials tested. Materials A and B had a main peak of number concentrations located between 0.01 and 0.1 μm, and a secondary peak between 0.15 and 0.4 μm (Figure 3a,c). The main number concentration peak for material C and for granite was located between 0.15 and 0.4 μm, and the secondary peak was between 0.01 and 0.1 μm (Figure 3e,g). The materials with higher silica content (A and B) had the main concentration peak in the nanometer range (<100 nm). Nanoparticles have been documented to translocate from the respiratory system to secondary target organs [44]. The nano-sized silica particles can migrate from the lungs to the bloodstream and reach other organs such as the liver and kidneys. Multiorgan accelerated silicosis has been described by Guarnieri et al. [45] who reported silica crystals in lung tissue and liver granulomas associated with accumulation of crystalline silica particles in the hepatic tissue of two workers exposed to quartz from cutting and polishing artificial kitchen countertops.

Mass concentrations were highest between 3 and 10 μm and above 20 μm for material A (Figure 3b). For material B, the main peak was located between 1.0 and 10 μm (Figure 3d). The main mass peak of material C was above 10 μm (Figure 3f) while for granite it was between 0.2 and 10 μm (Figure 3h).

### 3.2. Silica Concentrations

Table 3 provides a summary of the respirable dust and crystalline silica content collected on the PVC filters. The limit of detection (LOD) of the analysis was 0.005 mg for quartz, cristobalite, and tridymite. For respirable dust, the LOD was 0.05 mg. The field blank filters’ results were below the LOD and are not reported in Table 3.

Weight percentages of crystalline silica in Table 3 were calculated by dividing the mass of silica minerals per filter by the total respirable mass per filter. On average, materials A and B contained 51.5% and 53.7% quartz, respectively, indicating more than half of the weight percentage was silica. In contrast, the weight percentage of material C on average was 4.4% quartz, even lower than that of granite at 7.9%. Respirable dust concentrations averaged for three runs for each material were highest for granite at 430.4 mg/m^3^; more than 4-times higher than those of the other materials. For material A, the average respirable mass concentrations were 99.0 mg/m^3^; material B was at 88.8 mg/m^3^ and material C was at 82.6 mg/m^3^. A different pattern was observed for quartz concentrations; in this case, highest concentrations were measured for material A (50.8 mg/m^3^) closely followed by material B (47.7 mg/m^3^), then granite (33.46 mg/m^3^), and finally material C (3.7 mg/m^3^). Overall, material C presented the lowest respirable mass and quartz concentrations. While the trend for materials A, B, and C was similar to that reported by the DRIs in Figure 2, the respirable mass concentrations collected on filters for granite are inconsistent with the instrumentations.

The OSHA mineral dust permissible exposure limit (PEL) and exposure [46] for the three materials are calculated as follows:

PEL(respirable fraction)=10 ÷ (% quartz + (% cristobalite × 2)+(% tridymite × 2) + 2);(2)

(3) $$\mathrm{Exposure}=\frac{\sum_{i=1}^{n}\left(\frac{mg_{i}}{m_{i}^{3}}\times time_{i}\right)}{480\;min}$$

where *i* is the sample number and *n* is the total number of samples.

For material A, the PEL was 0.183 mg/m^3^, the exposure was 5662 mg/m^3^ and the severity (a unitless index calculated as exposure/PEL) was 30,982. For material B, the PEL was 0.176 mg/m^3^, exposure was 5082 mg/m^3^, and severity was 28906. For material C, the PEL was 1.29 mg/m^3^, exposure was 4724 mg/m^3^, and severity was 3716. For granite, the PEL was 0.926 mg/m^3^, exposure was 24,646 mg/m^3^, and severity was 26,651. A severity greater than 1 means exposure above the PEL. Respirable dust concentrations while grinding granite were at least 4-times higher than the other materials, however, exposure to respirable quartz was still at least 65% higher for materials A and B (Table 3). Other scientific entities have established stricter exposure limits due to the health effects of silica. For example, NIOSH established a recommended exposure limit time weighted average of 0.05 mg/m^3^; the American Conference of Governmental Industrial Hygienists (ACGIH) recommends an occupational exposure limit of 0.25 mg/m^3^. The proposed limit value in Europe is 0.1 mg/m^3^ [47]. The International Agency for Research on Cancer (IARC) classifies silica as a group 1 human carcinogen [48].

The exposure calculated for all four materials was extremely high. Our experiments were conducted inside a ventilated enclosure with a controlled environment, not a typical manufacturing facility scenario. Personal breathing zone concentrations in a manufacturing facility would be expected to be lower [49] than those measured inside the chamber. Our experiments, however, are comparable to work in confined spaces. These scenarios can occur in particular confined installations or when workers create temporary canopies or makeshift enclosures using plastic sheeting for performing on-site adjustments and cuts. During these types of installations, all of the materials tested would pose risks for acute silicosis.

Table 4 reports the analysis of the bulk particle debris collected during grinding. One set of bulk dust samples for each material was analyzed for total and respirable crystalline silica. As noted during the analysis of the respirable filters, materials A and B contained the highest concentrations of total crystalline silica at 91% by weight. These numbers are in line with the concentrations of quartz reported by the manufacturers in Table 1 (85–95% and >85%, respectively). Material C is also in agreement with the manufacturer specifications; we observed 9.6% total crystalline silica (Table 4) and the manufacturer reported <11% (Table 1). No manufacturer specifications were available for the granite sample and our analysis reported 30.7% silica content.

The CCSEM analysis of the bulk samples revealed that the weight percentage of respirable silica in particles smaller than 10 and 5 μm was highest in material A, followed by material B, then granite and finally material C (Table 4).

### 3.3. Microscopy Analysis

**Figure 4:**
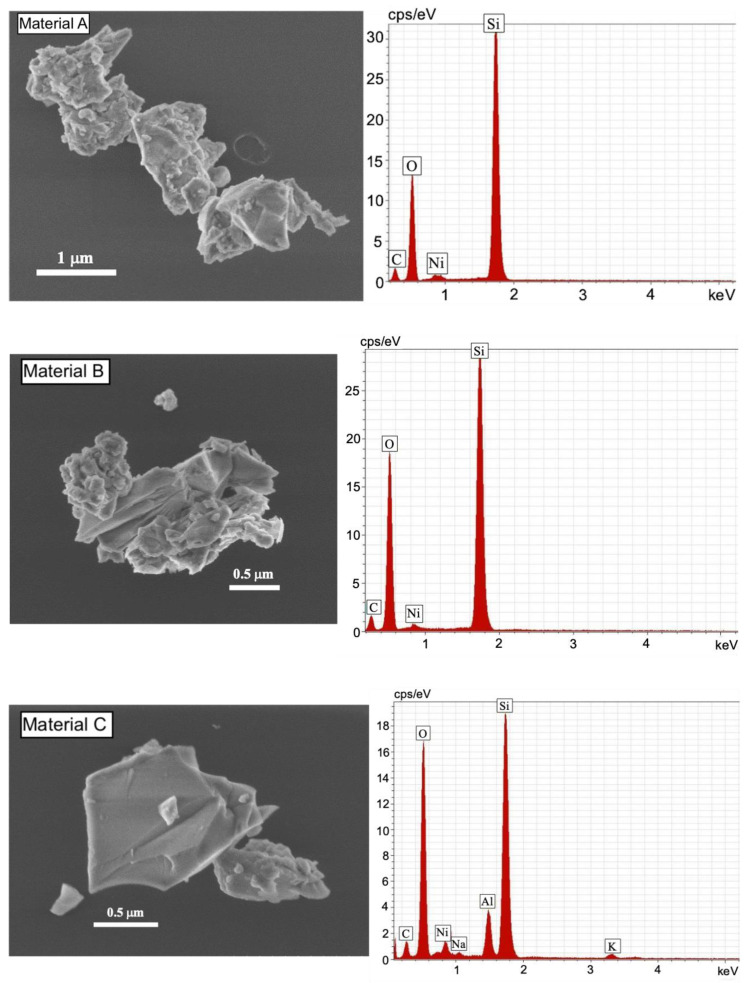

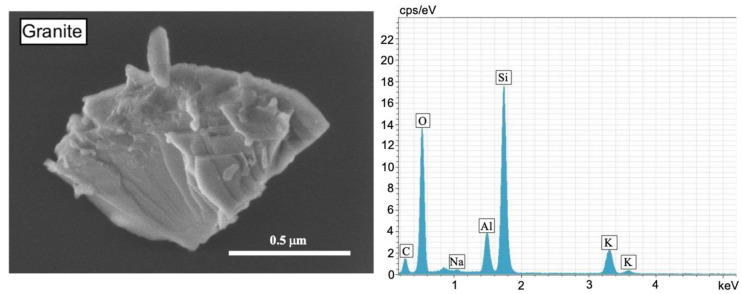
Secondary electron images of airborne grinding particles and energy dispersive spectroscopy analysis.

The results of the microscopy analysis of the TPS samples containing airborne particles collected directly on microscopy grids, are shown in Figure 4. Typical grinding particles present irregular shape and the chemical analysis revealed high counts of silica from materials A (35 cps) and B (29 cps), while low counts were reported from granite (18 cps) and material C (20 cps). 

## 4. Conclusions

Overall, the same pattern emerged from all of our analyses: the silica content of materials A and B was significantly higher than that of material C and granite. Although artificial countertop material C was harder to cut, requiring more force applied to the material by the operator of the angle grinder, its silica content was significantly lower than that of all other materials, including the natural granite sample. Artificial materials A and B may be easier to manipulate; however, the silica content being 3-times higher than the granite sample poses a substantial health risk associated with inhalation of aerosolized particles generated during manipulation processes similar to those we tested. Our results also suggest that the standard control measures used during manufacturing and installation processes (such as wet cutting) and use of personal protective equipment (such as respirators) are to be reevaluated. While wet cutting and use of respirators can be effective when properly and consistently implemented, the amount of dust generated during grinding and polishing creates a layer of dust debris that covers all surfaces surrounding the process. In manufacturing facilities, these deposited particles are subject to resuspension due to foot and forklift traffic when the materials are moved. It is unrealistic for workers to wear respirators and use dust suppression methods for an entire work shift. Even the most careful workers may be removing the respirators when moving around a workshop or when handling the materials, thus inhaling resuspended dust.

The granite sample generated at least 4-times higher respirable mass concentrations than the artificial materials while it contained 6-times less quartz by weight. The fact that artificial stone produces lower airborne dust concentrations indicates that using dust suppression methods that have proven effective during grinding of natural stone, could also be effective with artificial stone. The elevated silica concentrations of materials A and B, however, pose a high risk during dust resuspension when workers may not be wearing respirators. While resuspension with natural stone may have been ignored, it can no longer be neglected with artificial stones with high silica content such as materials A and B. 

It is our recommendation that manipulation processes involving the use of power tools for cutting, grinding, and polishing artificial stone countertops containing high concentrations of crystalline silica (i.e., above those of natural stones) include dust suppression both on the tools and on the work-stations. Aggressive engineering controls, such as the use of dust hoods/shrouds on the grinders, down-draft tables, and water jet sprays [50], should be designed and implemented for each specific operation to keep dust levels at a minimum, not only to avoid exposure during the manipulation process, but also to avoid dust resuspension when handling the materials and moving around the work environment. A floor drainage system should be used to clean the floors before the wet dust dries. We also recommend wearing respiratory protection and ventilating confined spaces, temporary hoods, canopies, or shrouds when cutting and grinding natural and artificial stone. 

This work demonstrated that for materials where the silica percentages are higher than natural granite, the dust formed during cutting and grinding contains high concentrations of nanoparticles and therefore has potential for greater impact on health, having effects not only on the respiratory system (e.g., acute silicosis), but also potential for translocation to other organs.

Plastic components have static charges that can attract highly charged particles generated during grinding processes. Conductive tubing was used to minimize this issue, however, there are limitations to their effectiveness and particle losses may still have occurred to the walls of sampling devices.

## Figures and Tables

**Table 1:** Sample materials’ composition and dimensions.

Material	Manufacturer Reported Composition	Sample Dimensions (Height × Width × Thickness)
A	Inorganic mineral fillers (85–95%) Polymerized polyester resin (5–15%) Additives and pigments (<5%)	21.6 × 35 × 1.95 cm
B	Quartz (>85%) Resin and color pigments (<15%)	10 × 10 × 0.6 cm
C	Aluminosilicates Silica (<11%) Zircon (depending on product) Inorganic pigments (<7%)	19 × 28 × 1.15 cm
Granite	Unspecified	29.5 × 29.5 × 2.7 cm

**Table 2:** Statistical Analysis Results.

Test	H₀	*p*-Value
ANOVA	All means are equal	<0.0001
Mood’s Median	All medians are equal	<0.0001
Kruskal–Wallis	All medians are equal	<0.0001

**Table 3:** Summary of average particle concentrations on polyvinyl chloride filters by number and mass and particle chemistry analysis by weight and number percent. StDev, standard deviation.

Material–Run	Weight Percentages of Crystalline Silica Minerals (%)			Airborne Dust Concentrations (mg/m^3^)			
	Quartz	Cristobalite	Tridymite	Respirable	Quartz	Cristobalite	Tridymite
A–1	52.8	<0.4	<0.4	78.9	41.7	<0.3	<0.3
A–2	48.6	<0.3	<0.3	106.7	51.8	<0.3	<0.3
A–3	53.0	<0.3	<0.3	111.4	59.0	<0.3	<0.3
Average	51.4	<0.3	<0.3	99.0	50.8	<0.3	<0.3
St Dev	2.5	0.1	0.1	17.6	8.7	0.0	0.0
B–1	55.3	<0.3	<0.3	87.4	48.4	<0.3	<0.3
B–2	51.4	<0.3	<0.3	94.3	48.5	<0.3	<0.3
B–3	54.4	<0.3	<0.3	84.9	46.1	<0.3	<0.3
Average	53.6	<0.3	<0.3	88.9	47.7	<0.3	<0.3
St Dev	2.0	0.0	0.0	4.9	1.3	0.0	0.0
C–1	4.9	<0.4	<0.4	77.3	3.8	<0.3	<0.3
C–2	4.0	<0.3	<0.3	90.5	3.7	<0.3	<0.3
C–3	4.4	<0.4	<0.4	80.0	3.5	<0.3	<0.3
Average	4.4	<0.4	<0.4	82.6	3.7	<0.3	<0.3
St Dev	0.5	0.1	0.1	7.0	0.1	0.0	0.0
Granite–1	8.6	<0.3	<0.3	356.4	30.6	<1.2	<1.2
Granite–2	7.9	<0.3	<0.3	420.3	33.3	<1.2	<1.2
Granite–3	7.1	<0.2	<0.2	514.6	36.5	<1.2	<1.2
Average	7.9	<0.3	<0.3	430.4	33.5	<1.2	<1.2
St Dev	0.8	0.1	0.1	79.6	2.9	0.0	0.0

**Table 4:** Total and respirable crystalline silica in bulk debris particles.

Sample	Total Crystalline Silica (Weight %)	Respirable Silica in Bulk <10 μm (Weight %)	Respirable Silica in Bulk <5 μm (Weight %)
Material A	90.8 ^§^	20.93	10.02
Material B	91.2 ^§^	14.16	5.48
Material C	9.6 *	1.79	0.58
Granite	30.7 *	5.25	2.71

^§^ no cristobalite or tridymite detected; * tridymite not detected. Cristobalite could not be determined due to the presence of feldspar.
